# Study on the Noise Reduction Characteristics of Porous Elastic Road Surface Based on Finite Element Analysis and Noise Field Tests

**DOI:** 10.3390/ma19081593

**Published:** 2026-04-15

**Authors:** Hongjin Liu, Zhendong Qian, Jinquan Zhang, Binfang Lan, Ke Zhong, Changhong Wang, Qi Wang, Xin Xu

**Affiliations:** 1 School of Transportation, Southeast University, Nanjing 211189, China; 2 China-Road Transportation Verification & Inspection Hi-Tech Co., Ltd., Beijing 100088, China; 3 Research Institute of Highway Ministry of Transport, Beijing 100088, China; 4 Taizhou Highway Industry Development Center, Taizhou 225309, China; 5 Shandong Hi-Speed Asphalt Co., Ltd., Jinan 250098, China

**Keywords:** elastic road surface, tire–road noise model, vibration noise, pumping noise, noise field tests

## Abstract

In order to study the noise reduction performance of Porous Elastic Road Surface (PERS), the vibration noise and air pumping noise has been separated from the tire–road noise through the finite element numerical simulation method. The tire–road noise model among the tire, road and surface air has been constructed by coupling of acoustic waves. The characteristics of tire–road noise under the PERS, Porous Asphalt Concrete (PAC), and Asphalt Concrete (AC) pavements have been analyzed through the modelling. The tire–road noise has also been investigated through the noise field tests. The generating process, coupling characteristics, and noise reduction performance of the vibration noise and the pumping noise of PERS pavements has been revealed. The results show that the tire–road noise was mainly generated by the vibration noise under the vehicle speed below 80 km/h. The proportion of pumping noise gradually exceeds that of vibration noise under the vehicle speed greater than 90 km/h. And the pumping noise gradually played the major role in the tire–road noise, which also increased with the increasing of vehicle speed. Comparing with AC and PAC pavements, PERS pavement exhibited the obvious advantages in noise reduction. Additionally, the reliability of the tire–road noise model has been verified through the field noise tests. It is expected that this work will serve as a reference for future research on the mechanics of the generation of tire–road noise, and try to provided theoretical support for the application of PERS.

## 1. Introduction

In recent years, the rapid development of urban transport has brought convenience to people’s lives, while the problem of traffic noise has become more prominent [1,2,3,4]. Traffic noise can be divided into two types: vehicle mechanical noise and tire–road coupled noise. The vehicle mechanical noise is the main noise source up to 35 km/h, but for higher speeds the propulsion noise is negligible over noise due to tire–road interaction. Additionally, improvements in vehicle manufacturing processes and technologies have greatly reduced the noise generated by the vehicle itself, making tire–road noise the most significant source of road traffic noise [5,6,7]. Methods to control traffic noise at low levels are generally from three aspects: at the source, along the sound path, and at the receiver. Therefore, some methods are used to reduce transport noise, such as adjusting the aggregate gradation of asphalt mixtures and installing sound barriers on both sides of the road [8]. It is worth noting that the low-noise pavement reduces noise from the source; it has the advantages of low cost and clear applications [9,10]. The PERS has been developed in Sweden in the late 70 s of the twentieth century [11]. PERS pavement, such as a low-noise pavement, is composed of stone and rubber particles as aggregate and polyurethane as the cementing agent. PERS pavement has the characteristics of porous sound absorption and elastic vibration absorption, making it one of the pavement materials with the best noise reduction effect [12,13]. Additionally, PERS pavement also has several functions, including drainage [14], self-stress de-icing [15], energy saving and carbon reduction, solid waste utilization, and so on [16].

The mechanism of tire–road noise has been studied gradually by many scholars through numerical simulation. Xu et al. [17] used computed tomography (CT) scan to gain images of cross sections of compacted Porous Asphalt Mixture (PAM) through X-rays, based on which a 3D model of the PAM structure is reconstructed, and assembled them into the coupled tire–pavement–air model. It can be observed from the noise sound pressure levels near the tire/pavement interface obtained from the finite element simulation that the tire/pavement noise curve shows obvious periodic peaks and valleys. Based on substantial experimental data collected for tire noise and pavement profile, Spies et al. [18] developed two artificial neural networks (ANN) to predict the tread pattern (ANN_TPN_) and the non-tread pattern related noise (ANN_NTPN_) components of tire noise, separately. The complete ANN Model of TPIN (AMOT) was capable of predicting TPIN well in narrowband, 1/3 octave band, and overall noise level for different tires on the pavement tested. Teti et al. [19] elaborated two models. The first model separates low and high frequency contributions, while the second one also considers noise around 1 kHz separately, using a three-band model. Both models are capable of forecasting the acoustic performance of newly laid low-noise road surfaces, using different road mixture parameters at different frequency ranges.

Existing studies on Porous Elastic Road Surface (PERS) have predominantly focused on material gradation optimization and mechanical performance enhancement, including mixture composition design [20], rubber particle content, PU binder types [21], effects of rubber thermal aging on durability [22], and other aspects. Some scholars have studied the noise reduction performance of PERS [23,24]. Ejsmont et al. [25] found that PERS pavements exhibited different noise reduction effects throughout the spectral range, and the noise reduction performance increased with the increase in speed. Gardziejczyk et al. [26], based on an experimental study on the acoustic properties of PERS pavements, concluded that the void ratio has a significant effect on the acoustic absorption coefficient of the PERS mixes.

Based on the overview of the literature about the research of PERS and the mechanism of tire–road noise, it is worth noting that the study of PERS has focused on the design of material gradation and road performance of mixtures. Few studies have been made on the mechanism of noise reduction in PERS, and these were mainly from the perspective of indoor tests. In addition, in the current research on the noise generation mechanism of tires and pavement, people usually take tires as the main research object and ignore the influence of pavement material properties on tire–road noise. The indoor and field noise tests are difficult to separate vibration noise and pumping noise from tire–road noise, so it is impossible to accurately analyze the composition of tire–road noise.

Therefore, the objective of this paper lies in exploring the noise reduction mechanism of PERS pavement. Based on the basic theory of spatial acoustic field and combined with the finite element numerical simulation method, the tire–road noise model among the tire, road, and surface air of PERS has been constructed in this paper. The model analyzes the spectral characteristics of vibration/pumping noise on PERS pavement and the influence of vehicle speed, and explains the weights of vibration noise and pumping noise in the coupling noise at different vehicle speeds. The relationship between pavement characteristic parameters and tire–road noise has been revealed, and the noise reduction performance of PERS pavement has been accurately assessed. The reliability of the model has been verified through the noise field tests. This study serves as a solid base of information to understand the mechanism of tire–road noise and provides the theoretical basis for its popularization and application.

## 2. Testing Method in the Field

**Figure 1:**
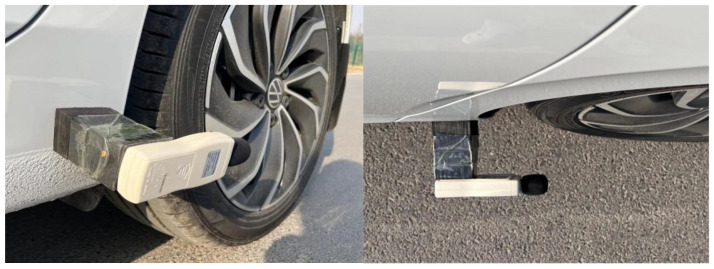
Near-field noise test noise meter layout.

**Figure 2:**
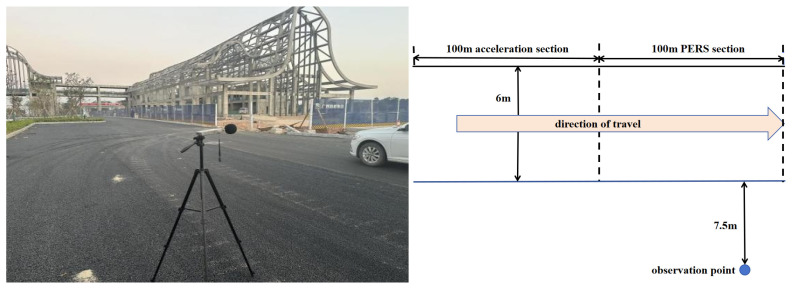
Far field noise test noise meter layout.

Far-field and near-field noise testing methods were used to conduct noise tests on the PERS-13 pavement test section paved in a project. In order to compare the noise reduction effect of PERS pavements, the noise of porous asphalt pavement PAC-13 and dense graded asphalt pavement AC-13 was also tested. The test vehicle is a front-drive light car, with a 100 km acceleration of 9.5 s. The unladen mass of the car to be tested is 1568 kg, and the tire model (195/65/R15, Hankook Tire, Seongnam-si, Republic of Korea) with a tread depth of not less than 1.6 mm, and the tire pressure meets the specified requirements. multifunctional sound level meter (AWA6228+, Hangzhou Aihua Instruments Co., Ltd., Hangzhou, Zhejiang, China) was used for the noise test. Noise test methods refer to Field Test Methods of Highway Subgrade and Pavement (JTG 3450-2019) [27], recording the maximum value of instantaneous traffic noise when the car to be tested passes through the test section at uniform speed under different speeds from 50 km/h to 100 km/h, the measurement was repeated three times, and the multifunctional sound level meter was laid out in the position as shown in Figure 1 and Figure 2.

## 3. Numerical Model

Based on the finite element software, a tire–road noise model among the tire, road, and surface air coupled with vibration noise and pumping noise was constructed. The model was divided into four parts: model geometry, material behavior, load type, and boundary conditions. Through the simulation calculation, the vibration/pumping noise sound field distribution maps of porous elastic road surface, the vibration/pumping noise data of different road surfaces were analyzed, and the vibration/pumping noise was analyzed by acoustic superposition.

### 3.1. Finite Element Component Modeling

The modelling of the tire–road noise model requires the construction of finite element components for the vibration noise model and the pumping noise model separately, including the tire components, the tire-ground contact model, the road surface model, and the air domain model. In the three-dimensional model components of the tire structure, the eight-node linear hexahedral cell (i.e., C3D8R cell) was selected as the grid cell form, and the radial tire of model 195/65/R15 was used as a reference for the modelling, with the tire radius and width taking the values of 0.2 m, and the tread thickness of 0.03 m. In the construction of the tire–ground contact model, the model was set up as a rubber block structure with external dimensions of 200 mm, 100 mm and 30 mm (length, width, and height), and the transverse pattern at the bottom of the rubber block with dimensions of 200 mm, 20 mm and 10 mm (length, width, and height). Pendant vibrations of a certain frequency and amplitude were used in the construction of the pavement model to represent the excitation of tires by wavelengths and amplitudes of different surface texture structures. Three types of pavement models, PERS, PAC, and AC, were constructed with structural dimensions of 1000 mm, 1000 mm, and 40 mm (length, width, and height), and the grids were constructed using C3D8R cells, with the approximate global size of each structural layer of the grid set to 0.1. The air domain of the vibration noise model was set up as a three-dimensional square with a side length of 1000 mm, and the pumping noise occurs only in the tire-contact area, so it was constructed for the air domain 0.2 m above the rubber block. The grid form using explicit acoustic cells uses four-node acoustic linear tetrahedral cells AC3D4, and the approximate global size of the air domain grid cells was set to 0.05.

### 3.2. Model Material Parameter Selection

In this paper, the Yeoh intrinsic model is chosen as the rubber model for tires, and the rubber material parameters are shown in Table 1.

The pavement panels were modelled with a linear elastic material model. According to JTG E20-2011 [28], the modulus parameters of the three pavement materials, PERS-13, PAC-13, and AC-13, were obtained by test under the action of 20 °C and 10 Hz loading frequency. The values correspond to the mechanical frequency response of a car travelling at room temperature at a speed of roughly 60–70 km/h. The pavement structural layer parameters are shown in Table 2.

In this paper, the density was set for the material properties of the air domain, and the bulk modulus was set under the acoustic unit of the gas type. The selected parameters are shown in Table 3.

### 3.3. Setting of Load and Boundary Conditions

In order to facilitate the application of displacement/turning angle boundary conditions and velocity/angular velocity boundary conditions on the tire structure, it is necessary to set the geometrical center of the tire structure as the reference point of the tire structure, and the coupling constraint state between the tread and the tire reference point.

**Figure 3:**
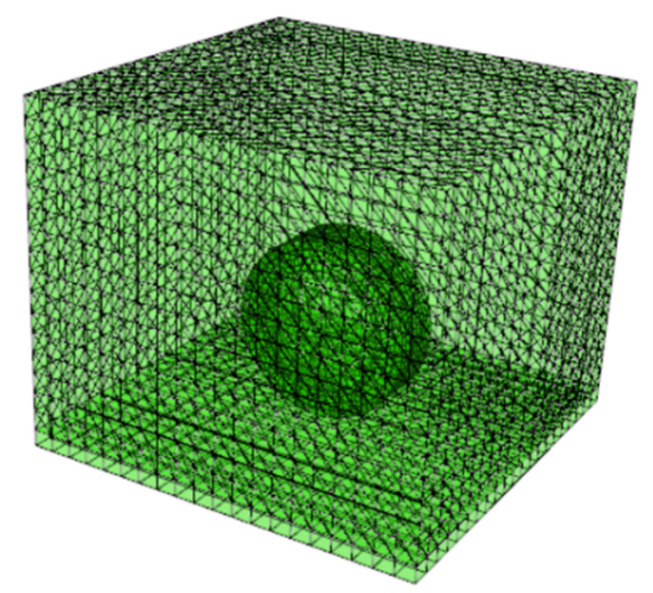
Vibration noise model.

In the vibration noise model, the initial rated gravity load applied to a single tire was set to 4000 N, the tire pressure was set to 0.25 MPa, and the amplitude of the tire vibration was set to 1 mm. The structural load of the tire was set by setting the amplitude curve and combining it with the gravity and the inflation pressure to complete the setup of the structural load of the tire. At the same time, a boundary condition with a vertical displacement of −0.001 was applied to the reference point at the geometric center of the tire to simulate the vertical excitation of the tire by the texture structure of the road surface. In order to rotate the tire, a boundary condition of rotational angular velocity was applied to the reference point, and the effect of vehicle speed on vibration noise was analyzed by varying the angular velocity of the tire. The initial angular velocity was set to 83.34 rad/s, which corresponds to a vehicle speed of 60 km/h. Meanwhile, the boundary conditions of no vertical, horizontal, and longitudinal displacements were set at the bottom of the road surface to achieve the purpose of fixing the road structure. The final vibration noise model among the tire, road, and surface air is shown in Figure 3.

The axle load and tire pressure in the grounded region of the tire and the vibration noise parameters were kept the same, and a quadratic function was used to fit the displacement-time curve to simulate the load applied to the rubber block pattern, which in turn characterizes the different travel speeds. The pumping noise model among the tire, road, and surface air was constructed. By modeling a single transverse tread groove, the road surface and the surrounding air domain, and loading the displacement-time curve on the upper surface of the tread block, the pumping noise was simulated and predicted according to the principle of acoustic-solid coupling. By varying the dynamic modulus of the road surface and the displacement-time condition imposed on the rubber blocks, the effects of road surface material and vehicle speed on the tire pumping noise were analyzed.

In the air model, tie constraints were used to define the contact relationship between tires and air to realize the coupled contact between tires, road surface, and air. In order to simulate the real situation, nonreflecting boundary conditions were set on the outer surface of the air domain, while acoustic resistance values were set on the contact surface between air and tire. The bottom of the air domain and the road surface, and the inner surface of the air domain and the outer surface of the tire were used in the bound constraint state.

## 4. Results and Discussion

### 4.1. Analysis of Simulation Results of Vibration Noise and Pumping Noise

**Figure 4:**
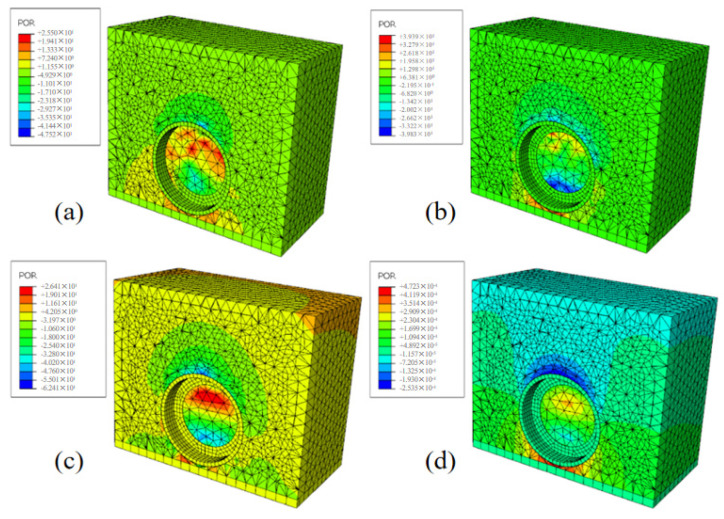
Vibration noise distribution cloud map (Y-Z section), (**a**) 0.025 s sound pressure distribution cloud map, (**b**) 0.05 s sound pressure distribution cloud map, (**c**) 0.075 s sound pressure distribution cloud map, (**d**) 0.1 s sound pressure distribution cloud map, respectively.

Reference point A (node 1023 of the model), which was 0.2 m away from the tire–road contact point, was selected to obtain the instantaneous change rule of sound pressure value caused by tire vibration at point A within 0.1 s. The vibration noise model was run among the tire, road, and surface air in its initial state to obtain the instantaneous sound pressure value change rule at point A caused by tire vibration when the tire speed was 60 km/h within 0.1 s. From Figure 4, it can be seen that the distribution cloud map of sound pressure fields at different times (0.025 s, 0.05 s, 0.075 s, and 0.1 s).

As can be seen from Figure 4, the sound is generated at the edge of the tire–pavement contact, and then the sound pressure propagates outward in an approximate semicircular diffusion, whereby the sound wave is propagated as a spherical wave in the spatial sound field. When the tire rotates, the air around the tire changes, resulting in a pressure difference, which is one of the reasons why the sound pressure propagates in a hemispherical shape. The presence of negative sound pressure values in the model is due to the direction of propagation of sound waves.

**Figure 5:**
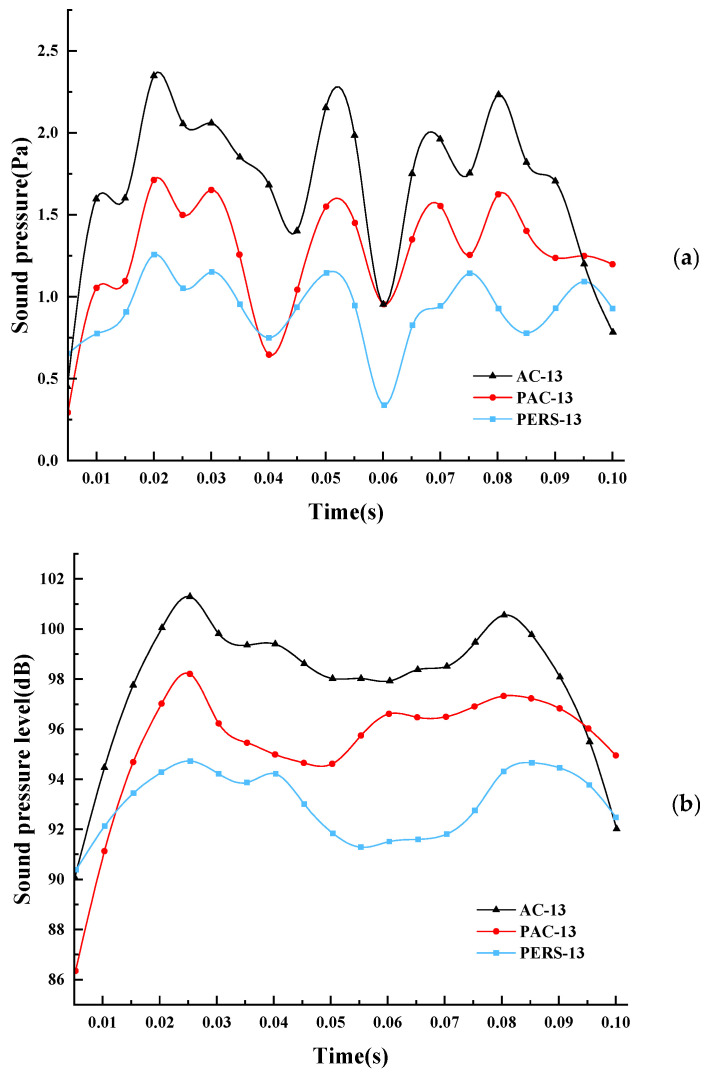
Vibration noise sound pressure and sound pressure level change at reference point A. (**a**) 60 km/h vibration noise sound pressure time domain curve, (**b**) 60 km/h vibration noise sound pressure level time domain curve.

By changing the road parameters, the sound pressure and sound pressure level change curves of reference point A in 0.1 s at 60 km/h speed state of AC-13, PAC-13, and PERS-13 were simulated, as shown in Figure 5. It can be seen that the vibration noise has peaks and valleys, and the curve has a certain periodicity, which was in line with the process of car tires colliding with the road surface at a certain frequency. On the whole, the vibration noise sound pressure of the PERS pavement is smaller than that of the PAC and AC pavements. It indicates that compared with the PAC and AC asphalt pavement, the PERS pavement mixes have a better attenuation, and the vibration and noise reduction performance of it is also better.

**Figure 6:**
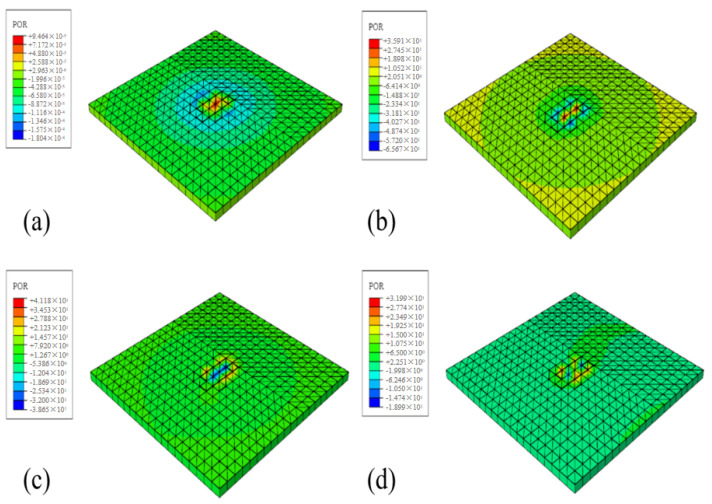
Pumping noise distribution cloud map (X-Y section), (**a**) 0.025 s sound pressure distribution cloud map, (**b**) 0.05 s sound pressure distribution cloud map, (**c**) 0.075 s sound pressure distribution cloud map, (**d**) 0.1 s sound pressure distribution cloud map, respectively.

Reference point B (node 835 of the model), which was 0.2 m away from the contact point between the rubber block and the road surface, was selected to obtain the change rule of the instantaneous sound pressure value caused by the pumping noise of the tire tread block at the junction in 0.1 s. After running the model, we obtained the distribution of pumping noise sound pressure at reference point B in the air domain at different moments within 0.1 s when the tire speed is 60 km/h, as shown in Figure 6. It can be observed that the pumping noise originates from the outer edge of the tire rubber block, and the sound waves propagate continuously in the form of spherical waves to the surrounding area. The sound pressure value gradually decreases with increasing distance from the sound source, which validates the model assumptions and indicates that the model has a certain level of reference value.

**Figure 7:**
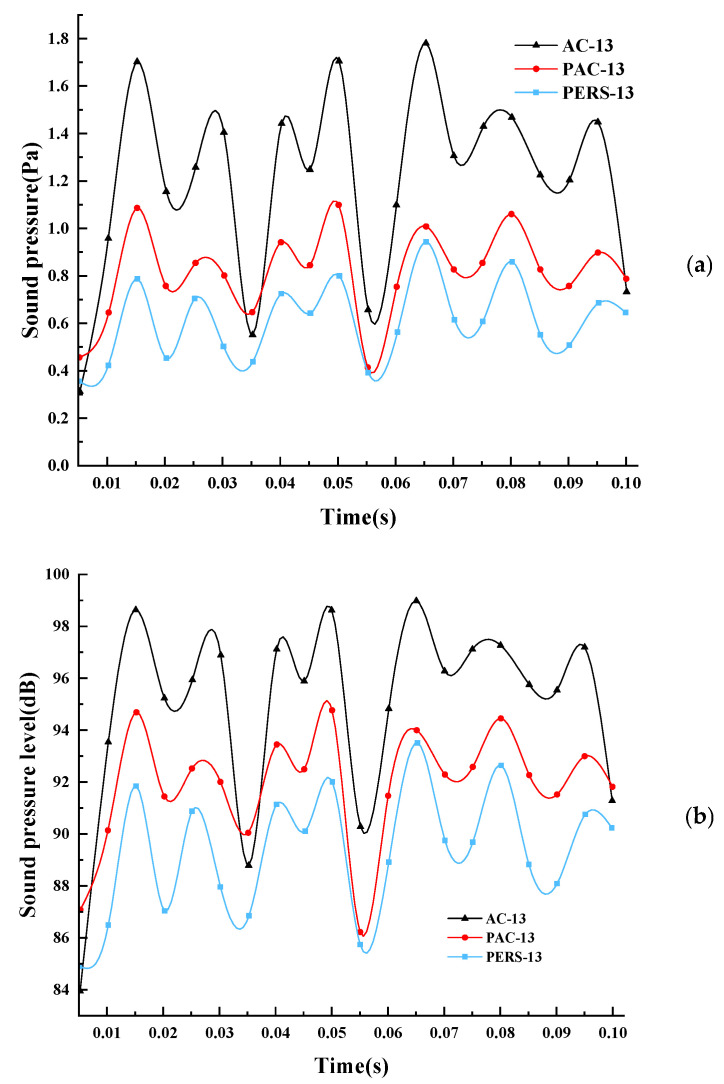
Sound pressure and sound pressure level change of pumping noise at reference point B. (**a**) 60 km/h pumping noise sound pressure time domain curve, (**b**) 60 km/h pumping noise sound pressure level time domain curve.

By altering the pavement parameters, the sound pressure and sound pressure level change curves of the pumping noise at the reference point B are simulated for the three types of pavements at different moments under the initial speed condition of 60 km/h, as shown in Figure 7. The sound pressure and sound pressure level of the pumping noise of the three types of road surfaces have obvious peaks and valleys, which reflect the process of compression and release in the pumping effect of the tread blocks. The extreme values of sound pressure appear near 0.015, 0.050, and 0.065, which are just at the time from compression to recovery of the tread block or the time stage of speed change. It can be seen that the process from compression to recovery of the tire tread block contributes the most to the pumping noise. Secondly, the moment of grounding of the tread block or the moment of speed change of the tread block can also cause the pumping noise of the tread block to a large extent.

**Figure 8:**
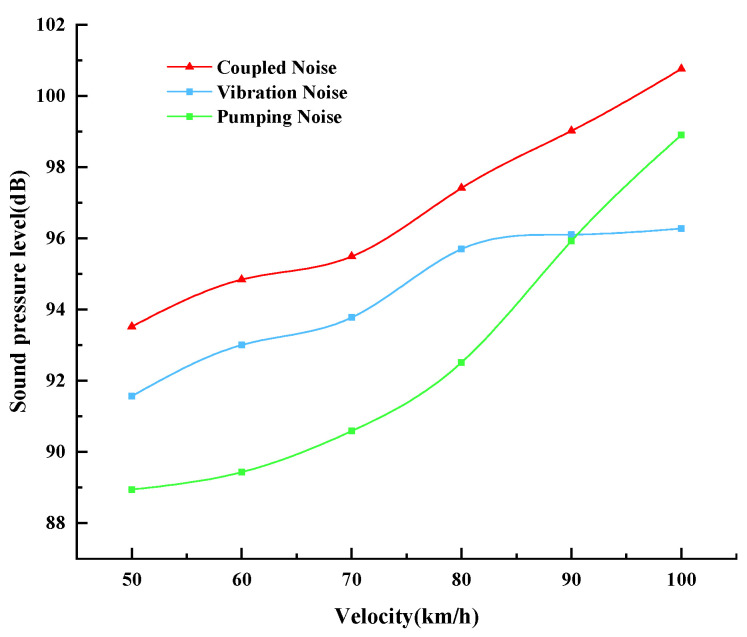
Vibration and pumping coupled noise relationship curve of porous elastic pavements.

The total sound pressure is calculated by superimposing the sound pressures of vibration noise and pumping noise through the method of sound wave superposition, and then the sound pressure level of the coupled noise is calculated. The relationship curves between the vibration and pumping noise sound pressure levels and the coupled noise sound pressure levels of porous elastic pavements under speed conditions from 50 km/h to 100 km/h are shown in Figure 8. As can be seen from the figure, the vibration noise increases with the increase in vehicle speed. This is due to the fact that the increase in vehicle speed leads to an increase in the contact frequency between the tire and the road surface, which in turn enhances the vibration effect of the tire excited by the road surface, thus enhancing the vibration noise. The increase in vehicle speed accelerates the process of squeezing and releasing air in the tire tread and road surface cavities, enhancing the pumping effect. It can be seen that the increase in vehicle speed accelerates both noise processes, thereby increasing tire–road noise.

When the car is running at low and medium speeds (below 80 km/h), the contribution of vibration noise to the tire–road noise is greater than that of pumping noise, i.e., at this time, the tire–road noise is dominated by the low-frequency vibration effect. As the vehicle speed further increases, it not only accelerates the vibration frequency between the tire and the road but also weakens the excitation effect of the road, causing the growth rate of vibration noise to slow down. When the vehicle speed is in the range of 70 km/h–90 km/h, the increase of pumping noise is obviously accelerated. When the speed reaches above 90 km/h, the pumping noise exceeds the vibration noise and becomes the main component of tire–road noise, which can be seen that the mechanism of tire–road noise is not the same at different running speeds. The tire–road noise is the result of the coupling of vibration noise and pumping noise, so it also tends to increase with the increase in vehicle speed. When the speed increases from 50 km/h to 100 km/h, the average increase in the tire–road noise of the porous elastic road surface can reach 7.24 dB.

**Figure 9:**
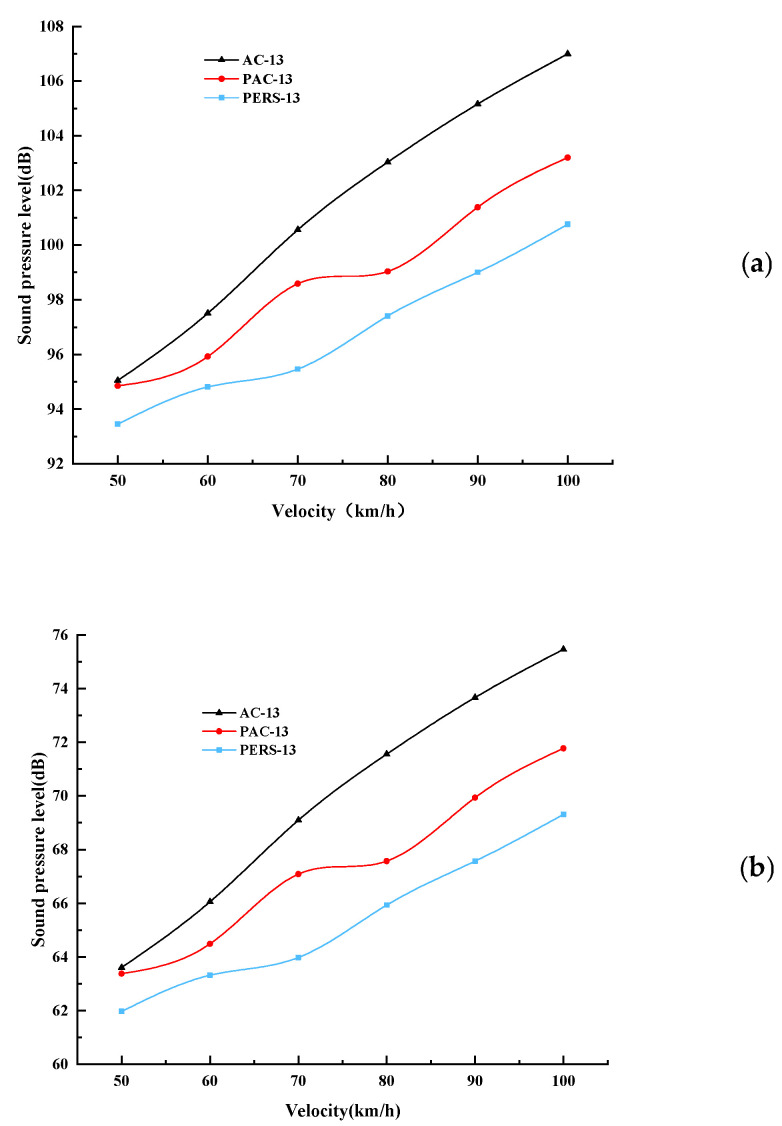
Comparison of sound pressure levels of near and far field noise under three types of pavements. (**a**) Near-field (0.2 m) noise sound pressure levels comparison, (**b**) far-field (7.5 m) noise sound pressure levels comparison.

After simulation, the near-field sound pressure levels and far-field sound pressure levels of AC, PAC, and PERS pavements under speed conditions ranging from 50 km/h to 100 km/h were shown in Figure 9. On the whole, compared with traditional pavement, PERS shows some noise reduction excellence at different speeds, especially in the middle and high speeds, with obvious advantages in noise reduction. The average value of tire–road noise of PERS is the lowest among the three types of pavements, and the average noise reduction is 2.5 dB less than that of PAC pavement, and 5.0 dB less than that of AC. The PAC pavement also shows a certain degree of noise reduction, which is due to the suppression of tire–road noise by the porous characteristic of PAC pavement itself. The noise reduction of AC pavement is the worst, and the main reason for this is that it is a dense graded asphalt pavement, whose void rate is small to weaken the pumping effect, which leads to the formation of larger decibel noise with increasing speed and more concentration of sound frequency. AC pavement has the worst noise reduction performance. The main reason is that it is a dense graded asphalt pavement, and its small void rate is not conducive to weakening the pumping effect. This leads to an increase in speed, the sound frequency is more centralized, and results in the formation of a larger decibel noise. It should be noted that when the speed is low, the difference between the tire–road noise of the three types of pavement is not significant, and the noise reduction characteristics of the pavement will be more obvious only in the middle and high speed cases.

### 4.2. Analysis of Noise Field Test Results

**Figure 10:**
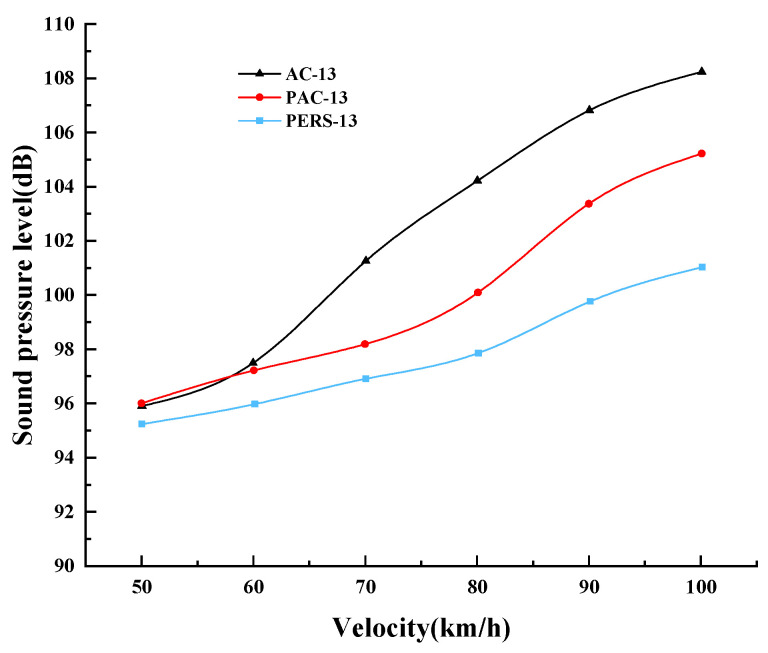
Near-field noise under different vehicle speed.

The 100 Hz~4000 Hz frequency band of 1/3 octave spectral noise test data was selected for data analysis, and it was found that when the vehicle speed was 60 km/h, the main composition of the measured near-field noise was the medium and low frequency vibration noise at 200 Hz~1000 Hz, and the peak center frequency of the sound pressure level of the tire–road noise at 60 km/h was 800 Hz. As the vehicle speed increases, the peak center frequencies at 80 km/h and 100 km/h are 1000 Hz and 1250 Hz, respectively, and the proportion of high-frequency noise in the tire noise increases. The near-field noise under different vehicle speed are shown in Figure 10. The near-field noise of all three road surfaces increases with vehicle speed, but the noise performance of each road surface is different. The sound pressure levels of the three road surfaces did not show significant differences at low speeds (vehicle speed ≤ 50 km/h). This is due to the tire–road noise at low speeds accounts for a relatively small proportion of the measured noise and is affected by the noise of the automobile engine powertrain. Thus, there is not significant difference in the level of each road surface noise at low speeds. The difference in the sound pressure level of the road surfaces is obviously larger when the vehicle speed is increased to 70 km/h and the differences among the road surfaces are more obvious with the increase of vehicle speed. This is not only in relation to the increase of vehicle speed, but also in relation to the increase of wind noise. Within the speed range of 50 km/h to 100 km/h, the average sound pressure level of near-field noise for PERS pavement is 2.29 dB lower than that for PAC pavement, and this value reaches 4.54 dB when compared with AC. This demonstrates the excellent noise reduction performance of PERS.

**Figure 11:**
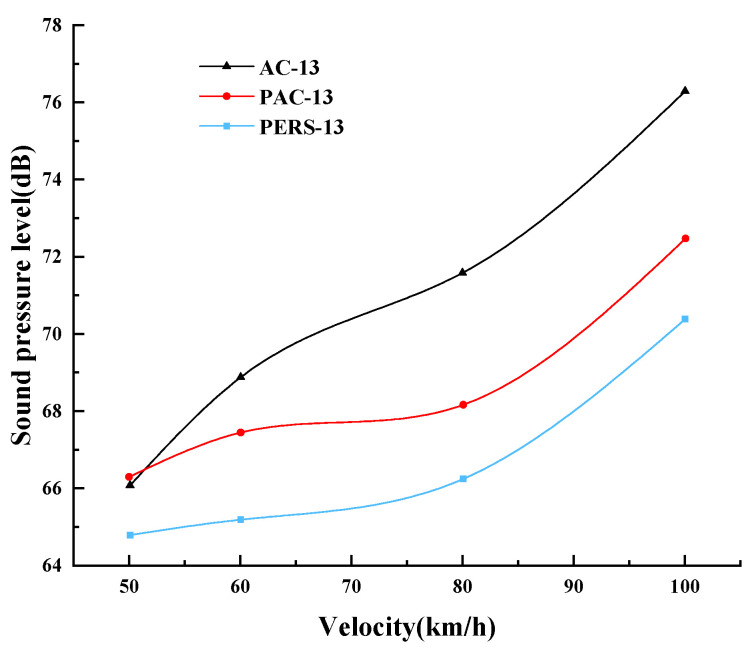
Far-field noise under different vehicle speed.

Similar to the near-field noise, when the vehicle speed is 60 km/h, the tire–road noise increases the frequency sound pressure level in the middle and low frequency bands, especially for the noise gain of 400~1600 Hz, and the peak center frequency is 1000 Hz respectively. The average results of the far-field noise of the three road surfaces under different speed conditions are shown in Figure 11. The far-field noise of the three road surfaces increases with the speed of the vehicle, and the tire–road noise sound pressure level increases to different degrees. When the vehicle speed is low (<50 km/h), the noise of the three road surfaces does not expresse the obvious difference. With the increase of vehicle speed, the traffic noise difference between the road surfaces becomes more obvious. When the vehicle speed reaches 100 km/h, the noise of PERS pavement is lower than that of PAC pavement and AC by 2.1 dB and 5.9 dB, respectively.

Compared with ordinary AC asphalt pavement, both PERS and PAC pavements have significant noise reduction effects, and the higher the vehicle speed, the more obvious the noise reduction effect of PERS and PAC pavements. The higher the speed, the stronger the vibration/pumping noise between tires and pavement. Both PERS and PAC pavements have a large void ratio and rich pore characteristics, which are conducive to reducing the air pumping effect and damping energy dissipation. The noise reduction effect of PERS pavement is further improved on the basis of PAC pavement. This is mainly due to the vibration-damping effect of rubber particles, thus, the PERS pavement has the maximum noise reduction performance.

### 4.3. Comparison of Noise Measurement and Simulation

**Figure 12:**
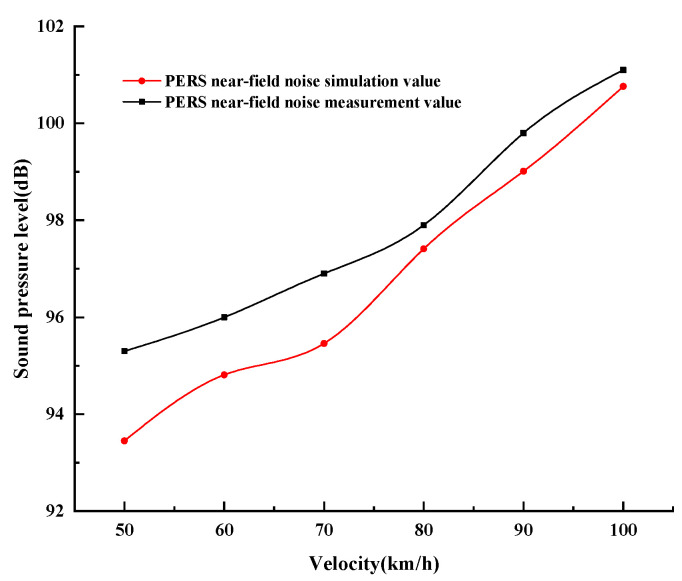
Comparison of near-field noise measurement and simulation results.

It can be seen from Figure 12 that the near-field noise of measured value is generally higher than that of the simulated value. This is mainly because the real situation is much more complicated than that in the simulation environment. At lower speeds, the tire–road noise will be affected by the mechanical vibration noise of the car’s engine and body parts. While at higher speeds it will be affected by the car’s wind noise and so on. However, the error value between the simulated mean value and the measured mean value of the near-field tire–road noise of the PERS pavement is within 1 dB, and the extreme difference between the two is 1.78 dB, which indicates that the finite element noise model of the PERS pavement can provide a reference for the prediction of the tire–road noise.

**Figure 13:**
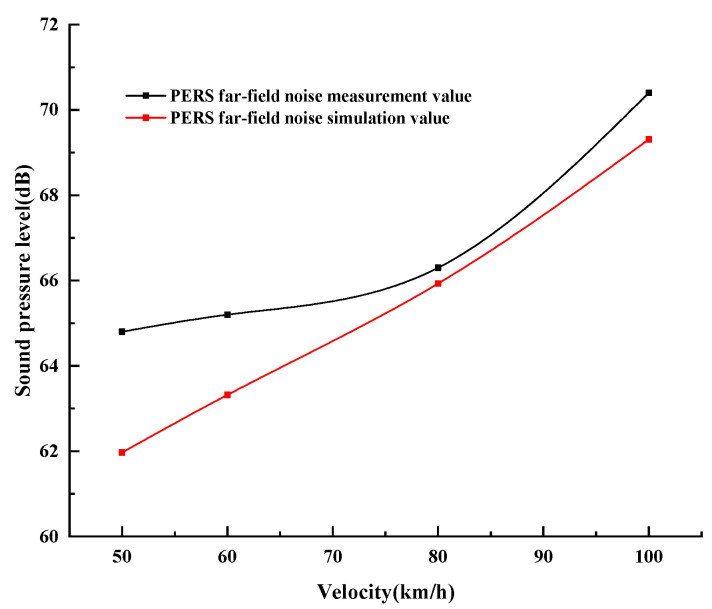
Comparison of PERS far-field noise measurement and simulation results.

The measured far-field noise data of PERS pavement is compared and analyzed with the simulation value, as shown in Figure 13. In the far-field noise test of PERS pavement, the measured noise values are slightly higher than the simulated values. This is because the impact of mechanical noise of the vehicle itself at low speeds during the test, and the impact of wind noise at high speeds, the existence of both leads to the measured value than the predicted value is large. This is consistent with the law of the near-field noise measurements and simulation values. Thus, the simulation results can also be used as a reference for evaluating the far-field noise of the PERS measurements.

## 5. Conclusions

Considering the coupled action of tire–road interaction, the tire–road noise models including the tire, road, and surface air of PERS, AC, and PAC have been constructed through ABAQUS. The distribution and transmission law of road vibration-pumping noise under different conditions has been studied. The tendency of road noise development under different vehicle speeds has been analyzed. The reliability of the model has been verified through the comparison with the noise field test results under different types of pavement. This study serves as a solid base of information to understand the mechanism of tire–road noise and holds great significance for the application of PERS. The main conclusions can be summarized as follows:

(1) The tire–road noise is composed of vibration noise and pumping noise together. The vibration noise is generated by the tire under the excitation of the road texture, and there are obvious peaks and valleys; the pumping noise is generated by the regular pumping and suction of the air cavity formed between the tire tread and the road surface. And there is obvious periodicity in the sound pressure curve.
(2) The vibration noise and pumping noise of the PERS increased with the increase in vehicle speed. The vibration noise is increased greatly at the speed under 80 km/h, while the pumping noise is increased greatly at the speed over 80 km/h. Among the tire–road noise components, the vibration noise accounts for a larger proportion of the tire–road noise under speeds below 90 km/h. When the speed increased continuously, the proportion of the pumping noise exceeded that of the vibration noise.
(3) Through the noise field tests, PERS had a better noise reduction effect than PAC and AC pavements under the same vehicle speed. At the vehicle speed of 100 km/h, the far-field noise of PERS was 2.1 dB lower than that of PAC pavement, and which also 5.9 dB lower than that of AC pavement.
(4) According to the comparative analysis of test results and simulation values, the error between the simulated value of PERS pavement tire noise and the measured value is relatively large in the medium and low channel road noise. The error value between the simulated and measured values of near-field tire path noise of PERS was within 1 dB. The measured values of far-field tire path noise were slightly higher than the simulated values. The tendency of noise development of tire–road noise model was consistent with that of the noise field tests, which verifies the reliability of the tire–road noise model.

## Figures and Tables

**Table 1:** Rubber material parameters.

Parameters	Parameters of the Yeoh Eigen Model						Density (kg/m^3^)
	C_10_	C_20_	C_30_	C_1_	D_2_	D_3_	
tread	0.70 × 10^6^	−0.27 × 10^6^	0.09 × 10^6^	7.25 × 10^−8^	0	0	1100
Sidewall	0.48 × 10^6^	−0.14 × 10^6^	0.39 × 10^6^	9.80 × 10^−8^	0	0	1050

**Table 2:** Setting of pavement structural layer parameters.

Pavement Type	Thicknesses (cm)	Modulus/MPa	Density (kg/m^3^)	Poisson Ratio	Damping Ratio
PERS	4	2505	1800	0.35	0.045
PAC		4248	2200	0.32	0.03
AC		6750	2400	0.3	0.03

**Table 3:** Air material parameters.

Material Type	Bulk Modulus/Pa	Density (kg/m^3^)
air	142,000	1.2

## Data Availability

The original contributions presented in this study are included in the article. Further inquiries can be directed to the corresponding author.
